# Prognostic biomarkers of renal cell carcinoma: Recent advances

**DOI:** 10.4103/0970-1591.38596

**Published:** 2008

**Authors:** Hiroshi Kitamura, Taiji Tsukamoto

**Affiliations:** Department of Urology, Sapporo Medical University School of Medicine, Sapporo, Japan

**Keywords:** Biomarker, prognosis, renal cell carcinoma

## Abstract

Recent advances in understanding the characteristics of renal cell carcinoma (RCC) have brought to our attention many prognostic markers that affect and predict the survival outcome of patients with the disease. For the moment, however, patients with RCC have not received any benefit from such markers. If a patient is diagnosed as “high risk” by using such prognostic markers, there is no promising systemic therapy available. In this review we mainly focus on biomarkers of RCC that can be applied for therapeutic use reported in recent publications. Several issues and limitations in the reported studies are also highlighted and discussed. Developing biomarkers from the viewpoint of therapeutic application will lead to improvement of the prognosis of RCC patients.

## INTRODUCTION

Renal cell carcinoma (RCC) is the most common malignant tumor of the kidney. It accounts for 3% of all adult malignancies and for approximately 95,000 deaths per year worldwide.[1] More than 30% of patients with RCC will develop metastatic progression after curative surgery. Many prognostic factors involving anatomical, histological and clinical aspects of the disease have been identified in RCC: the TNM staging system, tumor grade, sarcomatoid features, tumor size, performance status, etc.[2,3] However, no satisfactory treatment options exist for patients with advanced RCC at present and the response rate to immunotherapy using interferon-α (IFN-α) and/or interleukin-2 (IL-2) is less than 20%.[4] Therefore, urologists cannot improve the prognosis in the present situation, even though they can know such traditional factors of their patients. In this review, we focus on recent advances of prognostic biomarkers of RCC related to treatment, especially those that can be exploited as potential targets for immunotherapy.

## HYPOXIA-INDUCIBLE FACTORS

### Molecular pathways of hypoxia-inducible factors [Figure 1]

**Figure 1:**
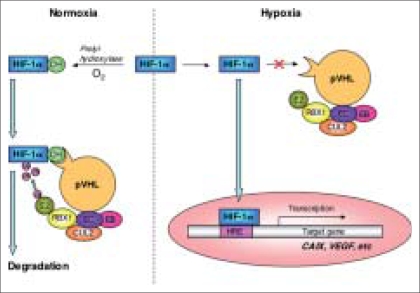
HIF pathway and target gene. Under normoxia, HIF-1α is hydroxylized by a prolyl hydroxylase and bound to pVHL, which forms a complex with elongine B (EB), elongine C (EC), CUL2, RBX1 and a ubiquitin-conjugating enzyme (E2). Then HIF-1α is ubiquitylated and degraded. In contrast, under hypoxia, non-hydroxylated HIF-1α enters the nucleus and activates target genes, CAIX, VEGF, etc. Genetic alterations of the VHL gene, lacking functional pVHL, cause high levels of HIF-1α in the same way

Somatic and epigenetic mutations of the von Hippel-Lindau (VHL) disease tumor suppressor gene are observed in 42-57% and 5-19% of sporadic clear cell RCCs, respectively.[5] In normoxic cells, hypoxia-inducible factor 1α (HIF-1α) is hydroxylated by specific prolyl hydroxylases, leading to recognition and binding by the VHL gene protein (pVHL) and targeting for ubiquitination and rapid degradation through the proteosome.[6] Under hypoxic conditions, HIF-1α is not hydroxylated and does not bind to pVHL, which leads to HIF-1α protein stabilization and translocation to the nucleus. In other words, hypoxia, like VHL mutations, induces upregulation of HIF-1α in patients with RCC, since lack of pVHL leads to reduced degradation of HIF-1α.[7] Hypoxia and VHL mutations also induce the expression of carbonic anhydrase IX (CAIX), a member of the carbonic anhydrase family that is thought to have a role in the regulation of cell proliferation in response to hypoxia and may be involved in oncogenesis and tumor progression.[8] CAIX is highly expressed in RCC and is considered a useful diagnostic biomarker.[9] Upregulation of HIF-1α is also associated with upregulation of vascular endothelial growth factor (VEGF), which promotes angiogenesis.[8]

### Hypoxia-inducible factor 1 α (HIF-1 α)

Lidgren *et al.*,[7] demonstrated that patients with conventional RCC having a high HIF-1α level survived significantly longer than those with low HIF-1α. Furthermore, multivariate analysis for conventional RCCs has revealed that HIF-1α is an independent prognostic factor for favorable prognosis. In conventional RCC, HIF-1α immunoreactivity is observed in cells throughout the tumor, consistent with HIF activation being caused by loss of VHL tumor suppressor function rather than microenvironmental hypoxia.[10] Therefore, these results confirmed the report of Yao *et al.*,[5] who demonstrated that RCC without VHL alteration had a poorer prognosis than RCC with VHL alteration.

### Carbonic anhydrase IX (CAIX)

Carbonic anhydrase IX is one of the most validated prognostic biomarkers of RCC. Bui *et al.*,[11] performed immunohistochemical analysis for CAIX expression on tissue microarrays from patients with conventional RCC. They demonstrated that 94% of the RCC tissues expressed CAIX and that decreased expression predicted a worse outcome for patients with locally advanced RCC and was an independent predictor of poor survival in patients with metastatic RCC. Kim *et al.*, showed that CAIX expression was a significant predictor in univariate and multivariate analyses and proposed accurate systems for predicting survival for patients with localized or metastatic RCC.[12,13] They concluded that prognostic models based primarily on protein expression profiles, including CAIX, could perform at least as well as TNM stage, histological grade or performance status. Atkins *et al.*,[14] reported that the level of CAIX in RCC tissues might also serve as a useful predictor of the response to IL-2 therapy and suggested that CAIX might maintain balanced pH in the tumor microenvironment, which might in turn be favorable for the maintenance of immune effector mechanisms. Meanwhile, Gilbert *et al.*,[15] detected CAIX expression in peripheral blood cells by reverse-transcriptase polymerase chain reaction (RT-PCR) and reported that the expression was associated with decreased disease-free survival in patients. Thus, CAIX is not only a prognostic biomarker but also a cancer-specific marker, although it can be expressed in normal tissues as well, including the gastric mucosa, large bile duct and pancreas.[15] Carbonic anhydrase IX-targeting therapy could be useful as a novel strategy for advanced RCC.

Recently CAIX-targeted therapies were reported in several studies. Uemura *et al.*,[16] discussed the results of a Phase I clinical study assessing the efficacy of CAIX-derived peptide vaccination in patients with cytokine-refractory metastatic RCC. Of the 23 HLA-A24-positive patients, three partial responses (PR) and six stable diseases (SD) were observed without severe side-effects. Bleumer *et al.*,[17] investigated vaccination with CAIX-peptide-loaded dendritic cells (DCs) in a Phase I clinical trial for patients with cytokine-refractory metastatic RCC. Unfortunately, however, there was no evidence for induction of CAIX-peptide-specific immunity and no clinical response. Lamers *et al.*,[18] transduced a single-chain antibody-type (scFv)-receptor based on murine monoclonal antibody G250, which recognizes an epitope on CAIX, into autologous T lymphocytes by using a retrovirus. They treated three patients with metastatic RCC with the scFv(G250)-transduced-T cells in combination with human recombinant IL-2 and observed specific cytolysis of CAIX^+^ target cells and production of IFN-γ on stimulation with such cells in all patients. However, liver enzyme disturbances reached National Cancer Institute Common Toxicity Criteria Grades 2 to 4 in all three patients. They performed liver biopsy, which suggested that the liver toxicity was caused by a specific attack of the scFv(G250)^+^ T-cells against CAIX^+^ bile duct epithelial cells. Therefore, CAIX may not be an appropriate target for specific therapy.

### Vascular endothelial growth factor (VEGF)

Upregulation of VEGF, the most potent growth factor for tumor vasculature, is significantly associated with upregulation of HIF-α.[8] Renal cell carcinoma is characterized by abundant neovascularization and metastases are more frequent in patients with highly vascularized primary RCC.[19] Thus it is hypothesized that VEGF is associated with tumor growth and progression. Although VEGF is the most potent proangiogenic protein described to date, with biologic effects relevant to tumor angiogenesis[20] and many clinical trials of VEGF-targeted therapy against advanced RCCs carried out,[21] the prognostic value is controversial in studies of its tissue[22–24] and serum[25] expression. However, sorafenib and sunitinib (tyrosine kinase inhibitors of VEGF receptors and platelet-derived growth factor) showed clinical benefits in Phase III trials for patients with metastatic RCC.[21]

## INHIBITOR OF APOPTOSIS PROTEIN AND APOPTOTIC PATHWAY

### Inhibitor of apoptosis protein (IAP) family

**Figure 2:**
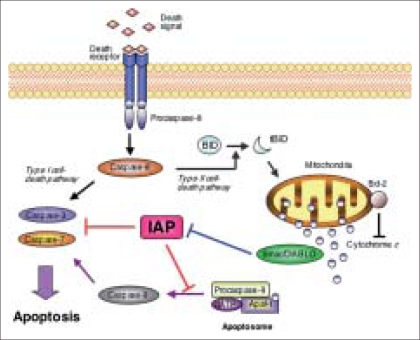
Apoptotic pathways and IAP. Of the two major pathways, IAP can block the intrinsic pathway by inhibiting terminal effector caspase-3 and caspase-7 and interfering with caspase-9 activity and processing. Smac/DIABLO acts as a dimer and contributes to caspase activation by sequestering IAPs

Apoptosis is essential to sculpt the developing organism by removing outdated or unneeded structures and also central to the homeostasis of adult tissues by maintaining the balance between cell production and cell elimination.[26] Since cancer cells acquire a longer lifespan by reducing apoptosis, apoptosis-related cancer-specific molecules can be expressed and be potential targets for specific therapies. There are two main apoptotic pathways [Figure 2]. The extrinsic pathway is triggered by the binding death receptor (CD95) and its ligands, which results in activation of upstream caspase-8. The intrinsic pathway is initiated by release of mitochondrial cytochrome *c* in the cytoplasm.[26] Cytochrome *c*, released by tBID (truncated BH3-interacting-domain death agonist) from the mitochondria, binds and activates apoptotic protease activating factor-1 (Apaf-1). It forms a multiprotein caspase-activating complex (apoptosome) and leads to activation of caspase-9, undergoing autoactivation to promote recruitment and cleavage of caspase 3. Caspase 3 cleaves its target substrates to affect the changes associated with apoptosis.[27]

Inhibitor of apoptosis protein can inhibit apoptosis by binding to prevent a common step downstream of mitochondrial cytochrome *c* release by inhibiting the terminal effector caspase-3 and caspase-7 and interfering with caspase-9 activity and processing. It has also been reported to affect cell division, cell cycle progression, signal transduction pathways and protein degradation.[28] Eight human IAPs have been identified. Of them, c-IAP1 (cellular IAP1), c-IAP2, XIAP (X-linked IAP), NAIP (neuronal apoptosis inhibitory protein) and BRUCE (BIR-repeat-containing ubiquitin-conjugating enzyme)/Apollon are expressed in normal adult tissues, whereas expression of survivin and livin/ML-IAP (melanoma IAP) is limited to tumor tissues. The expression of ILP2 (IAP-like protein 2) in normal or tumor tissue has not been investigated yet.

Although the expression of c-IAP1 and c-IAP2 is not tumor-specific, their overexpression can suppress chemotherapy-induced apoptosis *in vitro*.[29] Kempkensteffen *et al.*,[29] performed quantitative RT-PCR for c-IAP1 and c-IAP2 in 104 nephrectomized specimens and demonstrated that the tumor-to-normal ratio of c-IAP1 was a prognostic factor, especially in pT3 tumors. However, they also reported that 20% of the patients had lower c-IAP levels in cancer than in normal tissue, suggesting that c-IAPs cannot be a target of specific therapies. Ramp *et al.*,[29] reported that XIAP expression was found in 95% of conventional RCC by immunohistochemistry and the high-level expression was an independent prognostic parameter in conventional RCC in multivariate COX-regression analysis. However, XIAP cannot be a target for immunotherapy since convoluted tubules of non-neoplastic renal tissue show strong XIAP staining.

Survivin is overexpressed in various human malignancies and its expression is associated with features of biologically aggressive disease, resistance to therapy and poor clinical outcome in patients with various malignancies. Parker *et al.*,[30] demonstrated that approximately 30% of RCC patients had high levels of survivin expression, which was an independent predictor of conventional RCC progression and death from RCC. The five-year cancer-specific survival rates in patients with high and low survivin expression were 87% and 43%, respectively. Moreover, Byun *et al.*,[31] evaluated survivin expression in RCC tissues by using other criteria in which a positive finding was defined as staining of 10% or more of the cancer cells. They also demonstrated that survivin expression was an independent prognostic factor. The antiapoptotic activity of livin is more robust than that of survivin. We recently demonstrated that livin was positive in approximately 60% of RCC specimens, but the expression may not provide predictive information on survival.[32] However, livin may be recognized as a tumor antigen by the immune system in RCC patients because the anti-livin antibody levels in the sera of patients were significantly higher than those in healthy controls. Therefore, survivin-or livin-targeted therapy is a future possibility.

Smac (second mitochondria-derived activator of caspase)/DIABLO (direct inhibitor of apoptosis-binding protein with low PI) is a proapoptotic protein that in healthy cells resides in the intermembrane space in the mitochondria, but is released into the cytosol during apoptosis, where it interacts with IAPs and disrupts their ability to bind caspases [Figure 2]. The balance of Smac/DIABLO and IAPs determines the threshold for a variety of apoptotic stimuli. Mizutani *et al.*,[33] evaluated the level of smac/DIABLO by Western blot analysis for 78 RCC patients and demonstrated the high positivity (82%) of smac/DABLO expression in RCC and strong relationship between the expression and survival. Surprisingly, the five-year cancer-specific survival rates in patients with positive and negative smac/DIABLO expression were approximately 95% and 15%, respectively. Therefore, down-regulation of smac/DIABLO may be one of the most significant risk factors in survival of RCC patients. Unfortunately, however, smac/DIABLO cannot be a target for immunotherapy since it is expressed in a wide range of normal tissues.

## IMMUNOLOGIC MARKERS

### Tumor-infiltrating lymphocytes (TILs): Recent topics

It is well known that RCC frequently harbors numerous tumor-infiltrating lymphocytes (TILs), suggesting that a host antitumoral immune response is stimulated by the malignant transformation of cells. However, there is a paradoxical relation between increased levels of TILs and diminished cancer-specific survival.[34] Tumor-infiltrating lymphocytes were shown to be functionally defective, incompletely activated, depleted or anergic.[35]

Cózar *et al.*,[35] evaluated TILs of RCCs and found substantial numbers of natural killer (NK) cells and polarized Th1 CD4^+^ cells. Moreover, significantly fewer NK cells in peripheral blood, a lower proportion of CCR5/CXCR3/CD4^+^ cells and a higher proportion of CCR4/CD4^+^ cells were observed in patients with metastatic RCC in the study. These results indicate a change in helper T responses during the progression of RCCs. Donskov and von der Maase[36] analyzed potential prognostic factors in 120 patients with metastatic RCC who received IL-2-based immunotherapy. They demonstrated that the presence of intratumoral neutrophils and low numbers of tumor-infiltrating CD57^+^ cells (positive in a subpopulation of the total NK cells) were significant prognostic factors of poor survival both in univariate and multivariate analyses, whereas intratumoral macrophages, CD4^+^, CD8^+^, CD20^+^ and CD56^+^ cells were not significant ones. However, it is not yet clear why “low” CD57^+^ NK cells can be a prognostic factor for IL-2-based immunotherapy. Furthermore, there seems to be a discrepancy between the results of the two studies. Thus, the question remains: “Is infiltration of NK cells into tumors a positive prognostic factor or a negative one?”

Natural killer cells mainly kill tumor cells that have reduced major histocompatibility complex (MHC) Class I expression and can escape killing by cytotoxic T lymphocytes (CTLs). If the tumor cells have acquired escape mechanisms from CTLs, NK cell-infiltration can be a positive finding for suppressing the tumor. Therefore, it is important to evaluate the expression of MHC Class I molecules on RCC cells. We recently demonstrated that MHC Class I was down-regulated in 38% of conventional RCCs and the down-regulation was an independent prognostic factor.[4] Unfortunately, however, our study did not include many patients with metastatic RCC. It is necessary to evaluate the impact of MHC Class I down-regulation on responses to present immunotherapies. Thus far, the relationships among MHC Class I down-regulation, NK cell infiltration and survival have not been investigated.

Another topic of TIL is the regulatory T cell (Treg) that regulates the activation of other T cells and may be necessary to maintain peripheral tolerance to self antigens. One mechanism by which cancers evade immune destruction is by recruiting regulatory cells into the tumor microenvironment.[37] Treg is a small population of CD4^+^ T cells that coexpress CD25, the IL-2 receptor α-chain. In search of more specific markers for Treg, the transcription factor forkhead box P3 (FOXP3) has been identified. Forkhead box P3 is not only a key intracellular marker but also a crucial developmental and functional factor for Tregs. Siddiqui *et al.*,[37] demonstrated that the increased presence of CD4^+^ CD25^+^ FOXP3^−^ (but not CD4^+^ CD25^+^ FOXP3^+^) T cells was significantly associated with higher TNM stage, larger tumor size, the presence of coagulative tumor necrosis and poorer cancer-specific survival. Interestingly, they also showed that CD4^+^ CD25^+^ FOXP3^−^ TILs expressed more IL-10 (cytokine synthesis inhibitory factor) than CD4^+^ CD25^+^ FOXP3^+^ cells, suggesting that FOXP3^−^ Tregs have a powerful inhibitory function. Moreover, Dannuli *et al.*,[38] demonstrated that the elimination of Tregs using recombinant IL-2 diphtheria toxin conjugate DAB_389_ IL-2, acting like a CD25-specific antibody, reduced the number of Tregs present in the peripheral blood of metastatic RCC patients without severe side-effects and abrogated Treg-mediated immunosuppressive activity *in vivo*. They also demonstrated that the antitumor effects of DAB_389_ IL-2 followed by vaccination with RNA-transfected DCs significantly improved the tumor-specific T cell responses. Thus, depletion of Tregs is one of the strategies to suppress the progression of RCCs.

### Costimulatory ligand expression in RCC

The B7 family of molecules on antigen-presenting cells (APCs) is one of the best-defined costimulators for T cells. These molecules bind to the CD28 molecule on T cells and provide signals required for the activation of naive T cells. B7-H1, a member of the B7 family, can be induced on T lymphocytes, but aberrant expression on tumor cells has been described in various human malignancies. The expression of B7-H1 on tumor cells is considered to enhance apoptosis of activated tumor-specific T cells. Thompson *et al.*,[34] demonstrated that 66% of their RCC cases showed tumor-associated B7-H1 and elevated B7-H1 expression in RCC was significantly associated with high nuclear grade, the presence of coagulative tumor necrosis and poor prognosis. They also investigated B7-H4 and demonstrated that 59% of their RCC specimens exhibited staining for B7-H4, the expression of which was associated with poor prognosis.[38] Furthermore, B7-H1 and B7-H4 double positivity was a more powerful prognostic factor. The blockade of B7-H1 and B7-H4 by using monoclonal antibodies may be applied for therapeutic use, as Thompson *et al.*,[34] indicated in their preliminary experiment using murine RCC models.

## OTHER TOPICAL ISSUES

Here we will introduce two papers with high impacts on biomarker studies. Jiang *et al.*,[39] assessed the expression of insulin-like growth factor mRNA binding protein (IMP) 3 in RCC tissues by immunohistochemistry, quantitative PCR and Western blot analysis. IMP3 plays a role in RNA trafficking and stabilization, cell growth and cell migration during the early stages of embryogenesis. They demonstrated that IMP expression was strongly associated with high clinical stage, large tumor size and high tumor grade. Furthermore, the five-year overall survival was 82% in patients whose tumors did not express IMP3 versus 27% in those whose tumors expressed it and IMP was an independent prognostic factor. IMP3 is expressed in developing epithelium, muscle and placenta during early stages of embryogenesis, but is expressed at low or undetectable concentrations in adult tissues. Further studies of IMP3-targeted therapy are expected.

During the past decade, a large number of markers have been studied for their prognostic value in RCC. For example, molecular tumor proliferation markers, including Ki-67 (MIB-1), proliferation cell nuclear antigen (PCNA), topoisomerases and p100, have been investigated in many studies, but their value as prognostic markers is still controversial. Furthermore, urologists cannot apply most such results to improve the survival of RCC patients. Therefore, it is important to identify novel RCC-specific antigens. Recently, bioinformatical approaches have been used to search for such antigens. Yao *et al.*,[40] examined variations in the gene expression profiles of 33 RCCs and nine normal kidney tissues by using GeneChip microarrays and selected 149 genes highly overexpressed in conventional RCCs. Of them, they verified the overexpression of adipose differentiation-related protein (ADFP) in conventional RCCs by quantitative PCR. Furthermore, they stained the RCC specimens immunohistochemically and analyzed the relationship between ADFP expression and cancer-specific survival, demonstrating that high ADFP expression was an independent predictor of better outcome. Approaches like this should provide powerful tools for detecting novel cancer antigens that can be not only prognostic markers but also targets for specific immunotherapies.

## CONCLUSIONS

During the past decade, a large number of proteins that are putatively important in carcinogenesis and cancer biology have been studied for their prognostic value in RCC, but their clinical use remains controversial. Recently, however, novel biomarkers have been identified by various methods and some of them have been verified as clinical predictors of prognosis of RCC patients [Table 1]. It is important to identify potential markers for targeted biological therapies because randomized trials of traditional adjuvant therapies have brought no clinical benefit. Recent advances of powerful immunotherapies[18] suggest that markers expressed only in RCC cells are promising targets without severe side-effects. We need to continue investigation of such markers for improving prognoses of patients with RCC.

**Table 1:** Candidate markers for targeted therapy in this review

Marker	Prognostic value	Possibility as a target
Hypoxia inducible factors		
HIF-1α	Yes	Not investigated
CAIX	Yes	No?
VEGF	Yes	Yes
IAP family and related proteins		
c-IAP 1, c-IAP 2	Yes	No
XIAP	Yes	No
Survivin	Yes	Yes
Livin	No	Yes
Smac/DIABLO	Yes	No
Immunologic markers		
Regulatory T cell	Yes	Yes
B7-H 1, B7-H4	Yes	Yes
